# Exploratory Volatilome Profiling of Inflammation in Skin Fibroblasts: A Proof-of-Concept Study

**DOI:** 10.3390/ijms27083429

**Published:** 2026-04-11

**Authors:** Riccardo Di Stefano, Marco De Poli, Chiara Moltrasio, Angelo V. Marzano, Erika Rimondi, Elisabetta Melloni, Paola Secchiero, Giada Lodi, Marta Manfredini, Alberto Cavazzini, Annalisa Marcuzzi, Sergio Crovella, Flavio A. Franchina

**Affiliations:** 1 Department of Chemical, Pharmaceutical and Agricultural Sciences, University of Ferrara, 44121 Ferrara, Italy; riccardo.distefano@unife.it (R.D.S.); alberto.cavazzini@unife.it (A.C.);; 2 Dermatology Unit, Fondazione IRCCS Ca’ Granda Ospedale Maggiore Policlinico, 20122 Milan, Italy; chiara.moltrasio@policlinico.mi.it (C.M.); angelo.marzano@unimi.it (A.V.M.); 3 Department of Pathophysiology and Transplantation, Università Degli Studi di Milano, 20122 Milan, Italy; 4 Department of Translational Medicine and LTTA Centre, University of Ferrara, 44121 Ferrara, Italy; erika.rimondi@unife.it (E.R.); elisabetta.melloni@unife.it (E.M.); paola.secchiero@unife.it (P.S.); giada.lodi@unife.it (G.L.); 5 Department of Translational Medicine, University of Ferrara, 44121 Ferrara, Italy; marta.manfredini@unife.it (M.M.); annalisa.marcuzzi@unife.it (A.M.); 6 Council for Agricultural Research and Economics (CREA), 00184 Rome, Italy; 7 Department of Environmental and Prevention Sciences, University of Ferrara, 44121 Ferrara, Italy

**Keywords:** volatilomics, inflammation, fibroblasts, GC×GC-MS, SPME, VOCs

## Abstract

Inflammation is associated with metabolic alterations that can lead to the release of volatile organic compounds (VOCs) reflecting cellular biochemical activity. Profiling these volatile metabolites may provide insight into cellular responses to inflammatory stimuli, although their characterization in skin-derived cells remains limited. In this exploratory proof-of-concept study, we investigated the volatile metabolite profiles of human skin fibroblasts exposed to different inflammatory stimuli. Fibroblast cell lines were stimulated with polyinosinic:polycytidylic acid (Poly I:C), tumor necrosis factor-alpha (TNF-α), and lipopolysaccharide (LPS) to model viral-, cytokine-, and bacterial-associated stress conditions. Headspace solid-phase microextraction coupled with comprehensive two-dimensional gas chromatography and time-of-flight mass spectrometry (HS-SPME-GC×GC-TOFMS) was applied to analyze volatile metabolites released from the cell cultures, enabling exploratory profiling of the fibroblast volatilome. A data-processing workflow including pairwise comparisons between experimental groups and statistical filtering was implemented to identify volatile features associated with the different conditions. Several VOCs were tentatively identified, mainly belonging to alcohol, ester, and hydrocarbon classes, and showed differential abundance patterns between stimulated and control samples. Multivariate analysis indicated a separation between stimulated and non-stimulated groups, suggesting stimulus-associated differences in the volatile profiles of fibroblast cultures. While these observations may reflect metabolic responses occurring under inflammatory stimulation, the chemical identity and biochemical origins of several detected features remain to be confirmed. All in all, this study demonstrates the feasibility of applying HS-SPME-GC×GC-TOFMS-based volatilome profiling to investigate stimulus-associated changes in fibroblast cultures. The detected VOC patterns should therefore be considered preliminary observations requiring further chemical characterization and independent validation. Future studies including larger sample numbers, complementary biological verification of the inflammatory response, and more physiologically relevant experimental models will be necessary to further assess the robustness and potential relevance of these volatile signatures in the context of inflammatory processes.

## 1. Introduction

Dermal fibroblasts are increasingly recognised as heterogeneous and highly plastic stromal cells that actively orchestrate tissue homeostasis and inflammatory responses, rather than functioning solely as passive producers of extracellular matrix (ECM). Under physiological conditions, fibroblasts maintain ECM integrity, regulate tissue biomechanics, and support wound repair through coordinated mechano-transduction and crosstalk with keratinocytes, endothelial cells, and resident immune populations. These processes are metabolically demanding and tightly coupled to cellular redox control, positioning fibroblasts at the interface of inflammatory signalling, mechanical stress, and metabolic adaptation.

Single-cell transcriptomic and spatial profiling studies have profoundly reshaped the understanding of fibroblast diversity in human skin. Early analyses identified major fibroblast populations organised along transcriptional axes such as SFRP2/DPP4 and FMO1/LSP1, corresponding to distinct stromal programs with differential spatial localisation, secretory activity, and immunomodulatory potential [1,2]. Subsequent large-scale single-cell analyses across healthy and inflamed skin demonstrated that fibroblast states are dynamically reprogrammed in inflammatory microenvironments, where they contribute to immune cell recruitment, cytokine gradients, and tissue remodelling [3]. More recently, spatial genomics has further refined this framework by identifying immune-interacting fibroblast programs, including fibroblastic reticular cell-like and inflammatory myofibroblast-like states, highlighting fibroblasts as central integrators of immune cues, mechanical context, and metabolic stress [4].

Inflammatory activation of fibroblasts is accompanied by profound metabolic rewiring. Exposure to inflammatory stimuli induces shifts in glycolytic flux, fatty acid oxidation, mitochondrial function, and redox balance, frequently resulting in increased production of reactive oxygen species (ROS) [5,6]. These metabolic and oxidative processes regulate not only canonical soluble mediators such as cytokines and chemokines but also give rise to volatile organic compounds (VOCs) as by-products of altered cellular metabolism. In particular, ROS-driven lipid peroxidation of polyunsaturated fatty acids generates volatile alkanes (notably ethane and pentane) and volatile aldehydes (e.g., pentanal, hexanal), which have long been characterised as indicators of oxidative injury and inflammatory redox imbalance [7,8,9]. In parallel, inflammation-associated perturbations of mitochondrial metabolism and β-oxidation can influence the release of oxygenated VOCs, including ketones and short-chain aldehydes, expanding the volatilome associated with stromal activation [10].

Volatilomics, defined as the systematic profiling and characterization of VOCs released by living organisms, has emerged as a promising, minimally invasive approach for identifying novel biomarkers. In clinical research, particularly in oncology, breath and headspace VOC profiling has repeatedly demonstrated the ability to discriminate pathological from physiological states. Studies in lung and colorectal cancer cohorts have reported reproducible alterations in VOC classes such as alkanes, aldehydes, ketones, and aromatic compounds, reflecting tumour-associated oxidative stress, altered lipid metabolism, and mitochondrial dysfunction [11,12,13]. Importantly, mechanistic syntheses of cancer-associated VOCs explicitly link these chemical signatures to biochemical pathways including lipid peroxidation, cytochrome P450 activity, and metabolic reprogramming [14]. These pathways are not exclusive to malignant cells and are highly relevant to activated stromal fibroblasts exposed to inflammatory and oxidative stress, providing a biologically plausible bridge between fibroblast activation and VOC-based biomarkers.

Despite these advances, a key limitation of disease-oriented volatilomics remains the difficulty in attributing detected VOCs to specific cellular sources and metabolic pathways within complex tissues. VOC profiles measured in vivo integrate signals from multiple compartments, including epithelium, immune cells, stroma, microbiota, and environmental background, complicating mechanistic interpretation. Foundational studies have further demonstrated that classic oxidative stress-associated VOCs, such as breath pentane, are highly sensitive to sampling conditions and analytical confounders, underscoring the need for rigorously controlled experimental systems [15]. Notably, the specific contribution of skin fibroblasts, as metabolically adaptive and ROS-sensitive stromal hubs, to inflammation-associated volatilome signatures remains insufficiently defined.

To address this knowledge gap, controlled in vitro systems offer a reductionist framework to anchor volatilome changes to defined inflammatory and metabolic processes. Experimental studies indicate that inflammatory activation of fibroblasts induces metabolic shifts, including enhanced glycolysis, lipid oxidation, mitochondrial perturbation, and ROS production, all of which are mechanistically linked to VOC generation [16,17]. Accordingly, fibroblast-derived VOCs under inflammatory stimulation may provide sensitive and biologically interpretable readouts of immune–metabolic dysregulation.

In this proof-of-concept study, we explored the volatilomic response of primary human dermal fibroblasts exposed to defined inflammatory stimuli associated with distinct innate immune signaling pathways: polyinosinic:polycytidylic acid (Poly I:C), a synthetic analogue of viral double-stranded RNA, is commonly used to activate Toll-like receptor 3 (TLR3) and stimulate interferon-related inflammatory signaling [18]; tumour necrosis factor-α (TNF-α), a key mediator of inflammatory processes, which has been reported to promote fibroblast activation and cellular stress responses through Nuclear Factor-κB (NF-κB)-associated signaling pathways [19]; and lipopolysaccharide (LPS), a canonical Toll-like receptor 4 (TLR4) agonist, widely used to model bacterial inflammatory challenge and which has been associated with metabolic and oxidative responses in stromal cells [20]. Using comprehensive two-dimensional gas chromatography coupled to time-of-flight mass spectrometry (GC×GC-TOFMS), we analyzed volatile organic compounds released by fibroblasts following stimulation and explored differences in volatilomic patterns between stimulated and non-treated cells [21]. By examining volatilome variations in a controlled cellular model and its controls (treated vs. non-treated, treated vs. stimulus solution), this study provides an initial framework for investigating stimulus-associated volatile signatures in fibroblast cultures.

## 2. Results

### 2.1. Sample Group Characterization and Experimental Design

**Figure 1:**
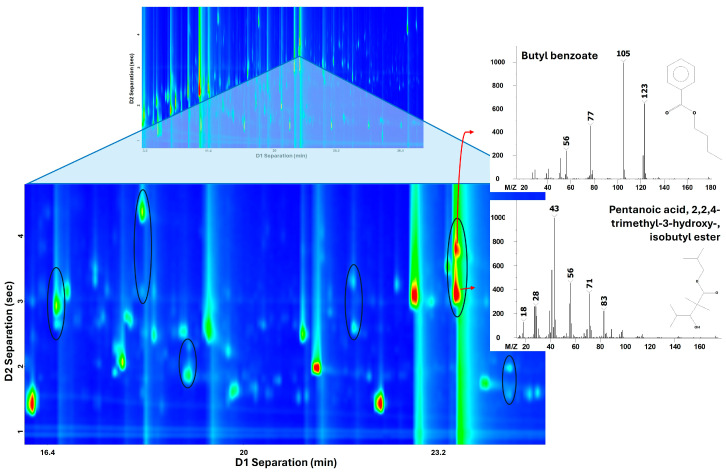
Section of a representative GC×GC-MS chromatogram highlighting an example of peak pairs separated along the second dimension.

Compared to conventional one-dimensional GC-MS, the GC×GC-MS technique provides enhanced separation capacity and reduces co-elution, which is particularly critical in the analysis of complex biological matrices such as volatile metabolomes [22,23]. Thanks to modulator signal enhancement, the structured two-dimensional separation, improved peak resolution and spectral deconvolution, a boost in both the detection and the identification of metabolites is achieved [24]. An example of analytes that are separated in the second dimension is shown in Figure 1, which represents a 2D plot from the cell culture control sample. Specifically, the circled regions represent some examples of successful 2D separation of peaks that would otherwise be coeluted in a single column. Despite the same retention time in the first dimension (*x*-axis), they undergo a differential retention mechanism into the second dimension, which results in different retention times in the *y*-axis.

To monitor analytical reproducibility and system stability, the internal standard (IS) response was monitored across all 29 chromatograms (Supplementary Figure S1). The coefficient of variation (CV) for the IS yielded a value of 7.3%. This low variability indicates satisfactory analytical repeatability and even though it does not represent a correction factor for biological variability, it is fundamental for the comparison of the chromatographic profiles across the sample set. Cell counts were also performed before aliquoting the supernatant for headspace analysis to ensure a comparable viability among the different treatments (Figure S2A,B).

Human dermal fibroblasts were selected as the in vitro model because they are now recognized as key immunomodulatory cells in cutaneous inflammation, actively shaping cytokine and chemokine networks within the skin microenvironment, as observed in the context of chronic inflammatory skin diseases [25], and therefore provide a cellular context for volatilomic experiments on inflammation-associated VOCs.

Based on a preliminary assessment of the cellular model, cell death-driven VOCs were excluded from evaluating cell viability and apoptosis (Supplementary Figure S2). The results indicated that at 6 h post-treatment, none of the treatments (Poly I:C, 25 µg/mL); TNF-α, 10 ng/mL; their combination; LPS, 25 µg/mL) reduced cell viability, with an average percentage of viable cells of approximately 97% (Supplementary Figure S2A). Moreover, apoptotic levels were comparable to those of the untreated control (Supplementary Figure S2B,C). Overall, these findings indicate that the treatments did not exert cytotoxic effects on the cultures under the experimental conditions tested.

We have also evaluated the ability of treatments to induce inflammation. The analysis revealed distinct cytokine response patterns depending on the applied stimulus. Interleukin-6 (IL-6) secretion was significantly increased following stimulation with TNF-α and with the combined TNF-α + Poly I:C treatment. Monocyte Chemoattractant Protein-1 (MCP-1) levels were significantly modulated by all tested stimuli, including LPS, Poly I:C, TNF-α, and TNF-α + Poly I:C. In contrast, interferon-gamma-induced protein 10 (IP-10) displayed a more selective response profile, with a significant increase observed only after stimulation with Poly I:C and TNF-α + Poly I:C. Overall, these findings demonstrate that NHDF-c cells exhibit differential cytokine secretion profiles in response to distinct inflammatory stimuli, highlighting stimulus-specific modulation of the inflammatory response (Supplementary Figure S3A).

The expression levels of *IL-6* and *interleukin-1β* (*IL-1β*) were also evaluated by quantitative PCR (qPCR) to further investigate their modulation following stimulation with Poly I:C and LPS. The results showed that the expression of both *IL-6* and *IL-1β* was modulated following treatments, indicating the activation of inflammatory signaling pathways in response to Poly I:C and LPS stimulation (Supplementary Figure S3B).

Regarding chemical analysis, the headspace of the sample supernatant was extracted *via* SPME and injected into the GC×GC-MS system. The resulting dataset containing the initial peaks or features was composed of the case group (i.e., inflamed cells), labelled as LPS, Poly I:C, TNF-α, and TNF-α + Poly I:C, and non-treated cells controls, labelled as NT. Moreover, additional controls were included in the experimental design, and specifically the (I) inflammation stimuli used to induce cell inflammation (i.e., the stimulus solution controls), the (II) culture medium only (i.e., the media controls), and (III) analytical system/fiber blanks (i.e., the analytical controls). Excluding the latter, case and control groups were prepared using the same harvesting conditions and analyzed using the same analytical conditions as described in Section 4.1, Section 4.4 and Section 4.5.

### 2.2. Data Analysis Workflow, Feature Selection, and Visualization

The resulting 29 HS-SPME-GC×GC-MS chromatograms were aligned based on the retention time and mass spectra of the analytes, yielding a data matrix composed of a total of 3090 initial peaks or features which were further processed.

**Figure 2:**
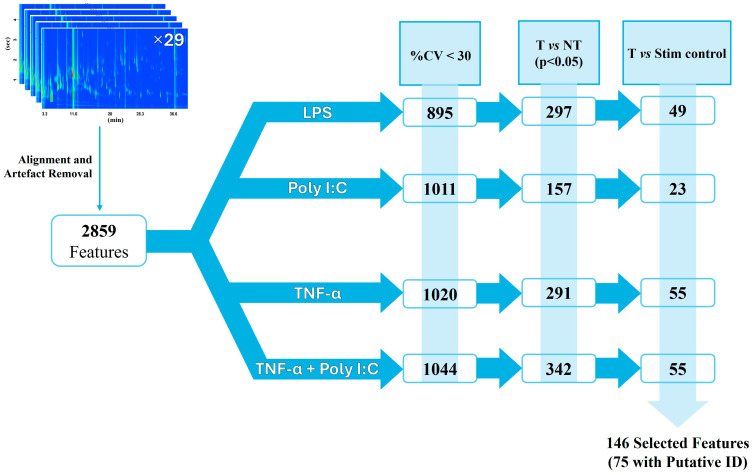
Workflow of data analysis reporting the number of features retained for each treatment group (T) after each filtering step (in the columns). After considering (1) the most reproducible features (CV < 30%), (2) the statistically significant features compared to its non-treated group (T vs. NT, Mann–Whitney U test), (3) the feature not originating from the corresponding stimulus solution control (T vs. Stim control), a total of 146 features, 75 of which were assigned tentative identities based on library matching and RI.

The data analysis workflow is shown in Figure 2: a first data cleaning step was performed to filter out the contributions from extraction fiber, the GC columns, or septa. This step resulted in a refined dataset of 2859 features, which were used for subsequent statistical analyses.

These refined features consisted of the contributions coming from the samples, both the cases (i.e., LPS, Poly I:C, TNF-α, TNF-α + Poly I:C) and the biological controls (i.e., NT, media, stimulus solution controls), thus additional selection strategies were necessary to highlight the metabolites characterizing the inflammation state. To focus on the most reproducible signals among biological replicates, only those with a coefficient of variation % (%CV) were retained. The %CV was calculated on the analytical response for all the features within each case group, and gave 895 fts in LPS, 1011 fts in Poly I:C, 1020 fts in TNF-α, and 1044 fts in TNF-α + Poly I:C, as illustrated in Figure 2.

At this point, to highlight treatment-specific differences in volatile metabolites, a series of pairwise comparisons (Mann–Whitney U test) were conducted between the not treated cells (NT) and each case group (i.e., LPS, Poly I:C, TNF-α, TNF-α + Poly I:C). This step reduced the number of features to 297 for LPS, 157 for Poly I:C, 342 for TNF-α, and 291 for TNF-α + Poly I:C. These lists of significant (*p* < 0.05) features for each case group, however, did not account for any potential interferences added during the inflammation treatment.

Since the case groups might carry volatile interferences originating from the inflammation-inducing solution and that can confound the statistical analysis, an additional pairwise comparison was introduced at this stage. It considered each case group with the respective stimulus solution control, and only the statistically significant (*p* < 0.05) features more abundant in the former were retained in the dataset, ensuring the focus was only on those metabolites originating from cellular responses.

This final list consisted of a total of 146 selected peaks. Specifically, 49 fts for LPS, 23 fts for Poly I:C, 55 fts for TNF-α, and 55 fts for TNF-α + Poly I:C, which were used for visualization and clustering analysis. Of these, 75 were tentatively identified based on library matching and RI, as reported in Section 4.4. A table reporting the 146 selected peaks along with their significance *p*-values, including retention times (1D and 2D), quantification ions, calculated and library retention indices (when available), spectral similarity scores, and the tentative annotations is reported in the Supplementary Materials (Table S1). Four additional tables, reported as Supplementary Tables S2–S5 (one for each treatment), show the corresponding log2fold change values of the selected feature for each treatment type against both the non-treated condition and the respective stimulus solution control. The impact of such selected features for the clustering of each treatment against the related controls is reported in the principal component analysis (PCA) score plots in Supplementary Figure S4a–d.

**Figure 3:**
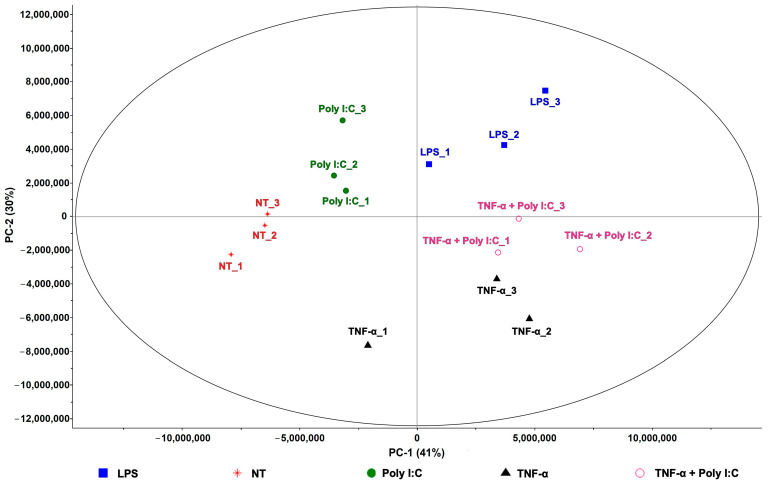
PCA score plot using selected features with a CV < 30% within each group to illustrate the distinct clustering of the experimental cell sample groups.

The cumulative effect of the selected peaks more reproducible among the groups was used to visualize the differential clustering among the treatments. The derived PCA score plot is shown in Figure 3 and the distinctive clustering of the cell groups can be observed, supporting the treatment-specific shifts in volatile metabolite patterns. Associated with this PCA score plot, the heatmap on the same features is reported as Supplementary Figure S5, showing the different distribution of the peaks among the sample groups.

### 2.3. Group Separation and Chemical Insights

**Figure 4:**
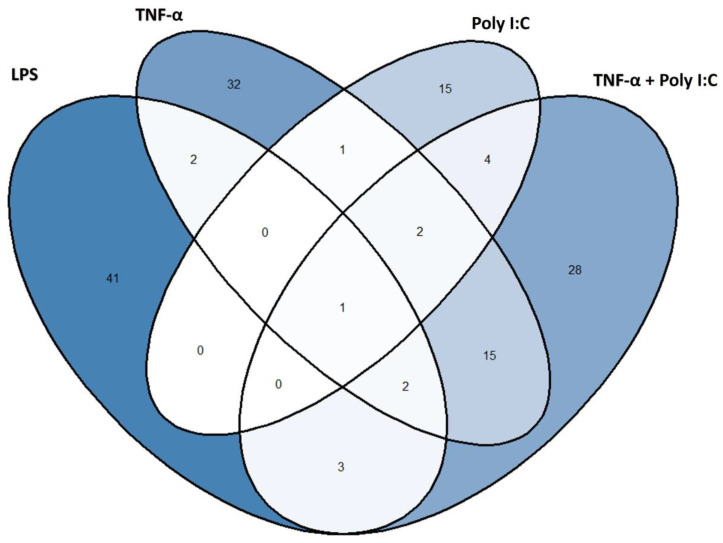
Venn diagram illustrating the unique and shared volatile features (tentative ID and unknowns) significantly altered by each inflammatory stimulus compared to untreated controls. The central intersection highlights one feature common to all four treatments (Unknown 55), suggesting a conserved metabolic response (reported in Figure 5).

A Venn diagram was constructed using the selected features across the four treatments (LPS, TNF-α, Poly I:C, and TNF-α + Poly I:C) and is shown in Figure 4. From the diagram can be highlighted both the treatment-specific and the shared volatile metabolites, potentially representing the inflammatory VOC response in fibroblasts.

**Figure 5:**
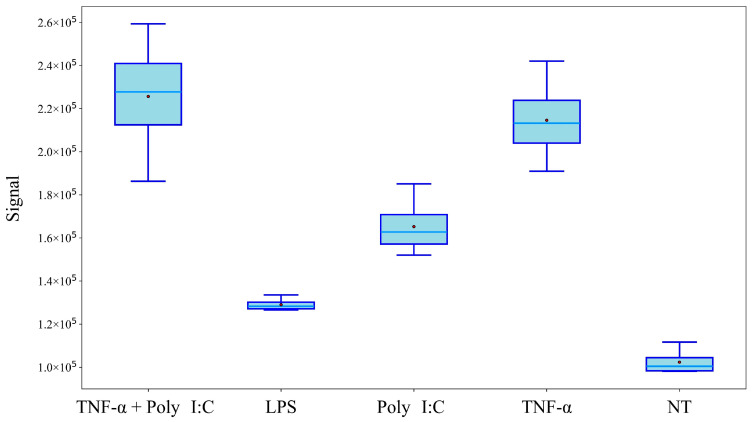
Box plot of the unknown feature (Unknown 55) that was significantly altered in all treatment groups compared to the NT. This feature is the same one highlighted as common to all inflammatory stimuli in the Venn diagram (Figure 4) and whose mass spectrum is shown in Supplementary Figure S6.

Among these, one feature was found to be significantly altered and in common to the four treatment groups compared to the NT condition. This molecule (labelled as Unknown 55) was not identified because of low mass spectral similarity when matching with mass spectral database; its mass spectrum is reported in Supplementary Figure S6. A box plot depicting its distribution across conditions is shown in Figure 5, further supporting its discriminative potential for inflammatory status. This compound is overexpressed, with log2fold change from 0.33 to 1.13 in LPS and TNF-α + Poly I:C treatment conditions, respectively.

**Figure 6:**
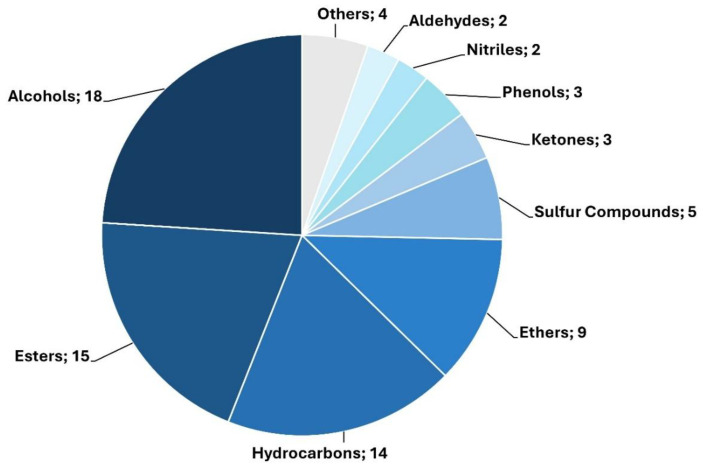
Relative abundance of putative chemical families for the 75 significant compounds that were assigned a tentative name. This classification provides a broad overview of the chemical nature of the key markers, revealing the main functional groups involved.

Also, the tentatively identified compounds could be grouped by putative chemical class, with a predominance of alcohols, esters, hydrocarbons, and ethers (Figure 6), observed among the treatment-specific VOC signatures. These chemical changes potentially suggest an active remodeling of fibroblast volatile metabolism under inflammatory stress.

## 3. Discussion

Fibroblasts, once thought of as structural components of the skin, are now increasingly recognized as active immunomodulatory cells with roles extending beyond matrix deposition and wound healing [26]. Their multifaceted capacity to sense inflammatory triggers, secrete cytokines, and interact with immune cells situate them as key orchestrators of cutaneous immune responses.

In our study, fibroblast exposure to stimuli mimicking viral-, cytokine-, and bacterial-associated inflammatory conditions was associated with differences in the emitted VOC profiles. These variations may reflect metabolic changes occurring under inflammatory stimulation; however, their biochemical origins and potential relevance remain to be further investigated.

An integrated analytical workflow has been established for non-targeted profiling of volatile metabolites in cell culture systems, with particular emphasis on correlating the metabolic response of human dermal fibroblasts with induced inflammatory stimuli [21]. The integration of HS-SPME-GC×GC-MS with a customized data processing framework constitutes a comprehensive platform for the identification of potentially relevant VOCs. The inclusion of matched stimulus solution controls was critical, as without this comparison, peaks originating from the added reagents could be misinterpreted as cell-derived metabolic responses. The applied filtering strategy therefore ensured that the retained peaks more specifically reflect the cellular response to inflammatory stimulation, rather than background or stimulus-related artifacts. In particular, since inflammatory stimuli added to the culture medium in the absence of cells act as combined controls for both medium- and stimulus-derived signals, they were used as the primary reference to rule out non-cellular contributions, as can be visually seen in the heatmap reported in Figure S5. Methodologically, this study provides a proof-of-concept framework for exploring volatile metabolic patterns in in vitro systems, illustrating how careful experimental design, inclusion of appropriate controls, and systematic data processing can support reproducible and interpretable analyses. While the current approach demonstrates the feasibility of capturing stimulus-associated volatilome changes, further optimization and validation in larger datasets and more physiologically relevant models will be needed to strengthen confidence in the observed patterns.

Despite the low number of biological replicates (*n* = 3), significant peaks (unadjusted *p*-values < 0.05) were obtained from the step-wise comparisons summarised in Figure 2, between T vs. NT and T vs. stimulus controls; these were used as a treatment-discriminating screening [27,28] to generate PCA visualization of Figure 3.

When looking at the results obtained in our experimental data set, the chemical classification of the tentatively identified VOCs revealed a consistent enrichment in alcohols, esters, hydrocarbons (alkanes and alkenes), and ethers, across all inflammatory stimuli applied to human dermal fibroblasts (Figure 6). This pattern reflects a stimulus-dependent but biochemically coherent response of fibroblasts to inflammatory stress, likely rooted in altered lipid metabolism, redox imbalance, and enzymatic remodeling.

Alcohols and esters were the most represented classes among the discriminant VOCs. Alcohols are frequently linked to oxidative stress and membrane lipid degradation, often arising as secondary products of lipid peroxidation or as intermediates in fatty acid metabolism. Their presence in inflamed fibroblast cultures supports the notion of ROS-mediated membrane remodeling—a well-documented feature of inflammation-induced cellular stress [29].

Esters, in turn, are synthesized through reactions between alcohols and fatty acids, a process mediated by alcohol acyltransferases and influenced by both enzymatic activity and substrate availability. The increased abundance of esters across all treatments may therefore indicate enhanced membrane phospholipid turnover, potentially driven by phospholipase A_2_ activation, and the subsequent availability of fatty acids and alcohols for esterification. This observation aligns with previous volatilomic studies in immune cells and cancer models, where ester accumulation has been associated with inflammatory activation and redox-driven metabolic shifts [30].

Hydrocarbons, particularly alkanes and alkenes, are recognized by-products of lipid peroxidation, generated through ROS-mediated cleavage of unsaturated lipids. These compounds have been detected in the exhaled breath of patients with systemic inflammation and in the volatile profiles of activated immune cells [15]. Their appearance in fibroblast VOCs further supports the oxidative stress landscape induced by inflammatory triggers such as TNF-α and LPS.

Ethers, though less abundant, also emerged as notable components of the inflammatory volatilome. Ethers may originate from both enzymatic processes and spontaneous reactions under oxidative conditions. Their role in cellular metabolism remains less defined, but prior work suggests that ether-containing lipid derivatives may act as modulators of inflammation and membrane structure [31].

Taken together, the observed chemical patterns suggest that fibroblast volatilomes are modulated by exposure to different inflammatory stimuli, with variations in VOC composition across alcohol, ester, and hydrocarbon classes. These differences appear stimulus-specific, highlighting distinct volatile features associated with each condition, while their underlying biochemical origins and potential functional significance remain to be further investigated.

The predominance of lipid-derived volatiles is consistent with fibroblasts’ role in extracellular matrix maintenance and membrane dynamics. Inflammatory activation induces profound metabolic remodeling in fibroblasts, producing distinct volatile signatures. In this study, the most enriched volatile compound classes included alcohols, esters, hydrocarbons (alkanes and alkenes), and ethers, indicating coordinated shifts in lipid metabolism and oxidative processes.

Specifically, phospholipase A_2_ (PLA_2_) activation during inflammation facilitates the breakdown of membrane phospholipids, releasing free fatty acids and short-chain alcohols, the primary substrates for ester biosynthesis via alcohol acyltransferases. Simultaneously, the inflammatory microenvironment elevates ROS, promoting lipid peroxidation and the generation of hydrocarbon volatiles. ROS-driven membrane damage may also yield either-containing lipids and their volatile derivatives through both enzymatic and non-enzymatic routes.

The convergence of increased precursor availability, redox imbalance, and altered enzyme expression supports the accumulation of these compound classes in the VOC profile.

Importantly, while most prior studies have focused on immune or tumor cell-derived VOCs, the present work highlights fibroblasts as contributors to the inflammatory volatilome, providing new insight into cell-type-specific volatile responses and laying the groundwork for further investigations into their functional and analytical significance.

Despite its promise, we acknowledge that our study has limitations. The in vitro fibroblast model enables controlled stimulation but lacks the complexity of the in vivo skin environment, where VOC production is shaped by interactions with keratinocytes, microbiota, immune cells, and vasculature. Thus, while the findings provide mechanistic insight, clinical extrapolation requires cautious validation. The exact biochemical origins of the identified VOCs remain to be unravelled. Their production may result from oxidative stress, lipid peroxidation, amino acid metabolism, or be influenced by mitochondrial or ER stress-necessitating metabolic tracing and enzyme activity assays. Discriminating between VOCs of cellular origin and exogenous contaminants (e.g., media-derived) is also essential for biomarker reliability.

To bridge the translational gap, in vivo validation using clinical skin samples, patient-derived fibroblasts, or murine inflammation models is needed. Intermediate models, such as inflamed skin explants or 3D reconstructed skin, may better reflect native tissue architecture while allowing controlled VOC analysis. Finally, functional validation should link specific VOCs to defined pathways, using strategies like gene silencing or overexpression (e.g., cyclooxygenases, aldehyde dehydrogenases) and isotopic flux tracing. Establishing causality between inflammation and VOC release is key to confirming their diagnostic relevance.

## 4. Materials and Methods

### 4.1. Cell Treatments and Harvesting

Primary Normal Human Dermal Fibroblasts (NHDFs) from juvenile foreskin were obtained from PromoCell (Heidelberg, Germany) and cultured in Fibroblast Basal Medium (PromoCell) with added Fibroblast Growth Medium SupplementPack (PromoCell). Cells were maintained at 37 °C in a humidified atmosphere with 5% CO_2_. Once confluent, cells were seeded into 6-well plates and allowed to adhere overnight. Cells were then exposed to the following inflammation-inducing treatments: lipopolysaccharide (LPS) 25 µg/mL (Merck, Darmstadt, Germany), polyinosinic:polycytidylic acid (Poly I:C) 25 µg/mL (InvivoGen, San Diego, CA, USA), tumor necrosis factor-alpha (TNF-α) 10 ng/mL (Cell Guidance Systems, St. Louis, MO, USA), and the co-treatment of TNF-α + Poly I:C or left untreated (NT). Treatment duration was standardized at 6 h with three biological replicates.

To ensure accurate interpretation of treatment-specific effects and exclude potential confounding signals unrelated to cellular metabolism, additional controls, such as the culture medium (media controls) and the inflammation stimuli used to induce cell inflammation (stimulus solution controls), were considered and prepared simultaneously under the same incubation conditions. These controls were processed alongside system/fiber blanks to assess any background contributions. 

### 4.2. Evaluation of Cell Viability and Apoptosis Induction

Cells treated for 6 h with Poly: IC (25 µg/mL), TNF-α (10 ng/mL), their combination, or LPS (25 µg/mL) were analyzed for viability by Trypan blue dye exclusion. Apoptosis cell percentage was evaluated by Annexin V-FITC/propidium iodide (PI) staining followed by flow cytometry analysis. After 6 h of treatments, cells were stained with Annexin V-FITC and propidium iodide using a commercial kit (Beckman Coulter, Brea, CA, USA), according to the manufacturer’s instructions. Samples were acquired with FACSCalibur flow cytometer (BD Biosciences, San Josè, CA, USA) and analyzed with software FlowJo, version 9.9.6 (Tree Star, Ashland, OR, USA). Data was processed using FlowJo software (BD Biosciences). Cell populations were identified based on Annexin V and PI fluorescence: viable cells (Annexin V^−^/PI^−^), early apoptotic cells (Annexin V^+^/PI^−^), late apoptotic cells (Annexin V^+^/PI^+^), and necrotic cells (Annexin V^−^/PI^+^).

### 4.3. Cytokine Profile Analysis

Cytokine concentrations were measured in supernatants of cells treated using the Bio-Plex Pro Human Cytokine 27-Plex Assay (Bio-Rad Laboratories, Inc., Hercules, CA, USA) according to the manufacturer’s instructions. Samples and standards were incubated with magnetic beads conjugated to specific antibodies against the target cytokines in 96-well plates. Data acquisition and analysis were performed using the Luminex xPONENT^®^ software, version 4.3, and cytokine concentrations were expressed in pg/mL.

From the same samples, total RNA was extracted using the RNeasy Mini Kit (QIAGEN, Hilden, Germany) according to the manufacturer’s instructions. cDNA synthesis was performed using a two-step reverse transcription (Thermo Fisher Scientific, Waltham, MA, USA), following the manufacturer’s protocol. Real-time quantitative PCR (qPCR) was then performed using the QuantStudio™ 3 Real-Time PCR Instrument (Thermo Fisher Scientific, Waltham, MA, USA). Gene expression levels were analyzed using specific primers and normalized to the housekeeping gene using the comparative Ct (2-DDCT) method.

### 4.4. Sample Collection and VOC Extraction

A volume of 0.8 mL of each cell culture supernatant was transferred under sterile conditions into 20 mL headspace vials, stored at −80 °C. Prior to extraction, 40% (%*w*/*v*) of potassium chloride (KCl) was added to each vial to induce a salting-out effect, enhancing VOC release into the headspace and improving extraction efficiency [32]. Volatile metabolites were extracted by headspace solid-phase microextraction (SPME), using a divinylbenzene/carboxen/polydimethylsiloxane (DVB/CAR/PDMS) fiber from Supelco (Bellefonte, PA, USA). The samples were thawed while maintaining the cold chain and placed on the autosampler rack at room temperature. Headspace vials were incubated and equilibrated under agitation for 10 min at 50 °C prior to fiber exposure and analyte extraction for 30 min at 50 °C. Before each sample extraction, the SPME fiber was pre-loaded with an internal standard (IS) to monitor instrumental stability. For IS preloading, a tridecane solution in dibutyl phthalate (10 ppm), was placed in a separate vial and pre-loaded (5 min at 50 °C) onto the SPME fiber before exposure to the sample headspace. The concentration and conditions for pre-loading were optimized based on preliminary experiments to achieve stable and reliable performance throughout the analytical batch [33,34]. Additionally, a standard mixture of linear n-alkanes (C7–C30) was analyzed under the same conditions as the biological samples to calculate retention indices (R.I.) for qualitative analysis.

### 4.5. GC×GC-MS Analysis

A Pegasus BT 4D (LECO Corporation, Mönchengladbach, Germany) GC×GC-TOFMS instrument with an Agilent 7890 GC (Agilent Technologies, Waldbronn, Germany) equipped with a dual-stage quad-jet cryogenic modulator (LECO Corporation) and an autosampler system for headspace analysis were used. The fiber was thermally desorbed into the GC injector for 1 min at 260 °C in split mode (1:5). The column set consisted in a non-polar phase Rxi-5SilMS (30 m × 0.25 mm id × 0.25 μm d_f_) connected to a mid-polar phase Rxi-17SilMS (2 m × 0.25 mm id × 0.25 μm d_f_) (Restek Corporation, Bellefonte, PA, USA). Helium was used as carrier gas at a flow rate of 1.5 mL/min. The primary oven temperature program was 40 °C (hold for 1 min), ramped to 250 °C at 4 °C/min. A fast temperature ramp of 15 °C/min to 300 °C ensured system cleaning for the subsequent analysis. The secondary oven offset was +5 °C, and the modulator offset was +15 °C relative to the primary oven. A modulation period of 4 s was used. A mass range of 40 to 500 *m*/*z* was collected at a rate of 150 spectra/s. The ion source was maintained at 250 °C. Instrument control and data acquisition was performed using ChromaTOF software 5.59.02 (LECO Corporation). All samples underwent the identical manipulation, sampling, and analysis conditions.

### 4.6. Data Processing and Statistical Analysis

Raw data were processed on ChromaTOF Sync software v2.0.15.0 (LECO Corporation) integrating peak detection, spectral deconvolution, and peaks alignment across all samples. From the alignment, a list of peaks was obtained and subjected to data curation (background subtraction and removal of known artifacts) and statistical analysis. Missing values were replaced with half of the minimum detected signal across the dataset. A filtering step based on the Coefficient of Variation (CV < 30%) was first applied to each treated cell group (LPS, TNF-α, Poly I:C, TNF-α + Poly I:C) to retain the most reproducible features. The retained peaks were then subjected to pairwise statistical comparisons performed using the Mann–Whitney U test implemented in R (version 4.5.1, http://www.R-project.org/, accessed on 20 January 2026) using the Wilcox-test function, before between each treatment cell group and the untreated cell control (NT). The resulting peaks were further compared (Mann–Whitney U test using the Wilcox-test function) against the corresponding stimulus solution control. *p*-values were calculated using the asymptotic approximation without continuity correction and were used as an initial feature-screening criterion [35,36]. No data normalization was applied prior to univariate analysis, as comparisons were performed on a feature-wise basis. The final list of significantly different features was obtained, and raw signals were mean-centered and subjected to PCA for visualizing sample groups clustering. PCA was carried out using The Unscrambler X software (CAMO software AS, version 10.4, Oslo, Norway). Orange Data Mining software (version 3.39.0, Bioinformatics Lab, University of Ljubljana, Slovenia) was used to create the heatmap in Supplementary Information.

For putative identification, the selected peaks were subjected to NIST 2023 spectral database and retention index (RI) matching. Specifically, putative names were assigned to peaks with >70% similarity and 20 RI tolerance agreement (when available) compared to the library.

## 5. Conclusions

The observed dysregulation of alcohols, esters, hydrocarbons, and ethers across different inflammatory stimuli in fibroblast cultures highlights reproducible changes in volatile metabolite patterns associated with cellular responses to stimulation. These volatile features, captured directly from cell culture supernatants, may reflect underlying metabolic alterations, though their specific biochemical origins remain to be confirmed. This proof-of-concept study demonstrates the feasibility of fibroblast-based volatilomics as an exploratory methodological platform for characterizing stimulus-associated volatile organic compound profiles. VOC analysis provides a minimally perturbative readout of cellular metabolic activity under controlled conditions, allowing for longitudinal monitoring of responses without repeated invasive sampling.

The methodological framework presented here, integrating standardized inflammatory stimulation with biological controls, comprehensive two-dimensional gas chromatography–mass spectrometry (GC×GC-MS), and multistep statistical filtering, offers a reproducible approach for detecting and evaluating VOC contributions. This workflow is not limited to a single cell type or stimulus and can be adapted to other skin-relevant cell models and inflammatory conditions.

By establishing a scalable and generalizable analytical pipeline, this study provides an in vitro foundation for the exploratory investigation of VOC patterns across diverse inflammatory contexts and for guiding future studies aimed at chemical characterization and biological validation of these volatile features.

## Data Availability

The data supporting the findings of this study are available from the corresponding author upon request. Due to the high-dimensional and proprietary nature of the GC×GC-TOFMS volatilomic datasets, raw chromatograms and processed feature matrices are not publicly archived but can be shared for academic research purposes under data use agreements.

## Supplementary Materials

The following supporting information can be downloaded at: https://www.mdpi.com/article/10.3390/ijms27083429/s1.
