# Supplementary material for: Exploratory Volatilome Profiling of Inflammation in Skin Fibroblasts: A Proof-of-Concept Study
Source: Int J Mol Sci. 2026 Apr 11;27(8):3429. doi: 10.3390/ijms27083429 (PMC13116231; doi:10.3390/ijms27083429)
Supplement: Supplementary file 1 [file ijms-27-03429-s001.zip › ijms-4192286-supplementary.pdf]

Supplementary Information

Exploratory Volatilome Profiling of Inflammation in Skin Fibroblasts: A Proof-of-Concept Study

Riccardo Di Stefano [1], Marco De Poli [1], Chiara Moltrasio [2], Angelo V. Marzano [2,3], Erika Rimondi [4], Elisabetta Melloni [4], Paola Secchiero [4], Giada Lodi [4], Marta Manfredini [5], Alberto Cavazzini [1,6], Annalisa Marcuzzi [5], Sergio Crovella [7,*] and Flavio A. Franchina [1]

[1] Department of Chemical, Pharmaceutical, and Agricultural Sciences, University of Ferrara, 44121 Ferrara, Italy; riccardo.distefano@unife.it (R.D.S.); marco.depoli@unife.it (M.D.P.); alberto.cavazzini@unife.it (A.C.); flavioantonio.franchina@unife.it (F.A.F.)
[2] Dermatology Unit, Fondazione IRCCS Ca' Granda Ospedale Maggiore Policlinico, 20122 Milan, Italy; chiara.moltrasio@policlinico.mi.it (C.M.); angelo.marzano@unimi.it (A.V.M.)
[3] Department of Pathophysiology and Transplantation, Università Degli Studi di Milano, 20122 Milan, Italy
[4] Department of Translational Medicine and LTTA Centre, University of Ferrara, 44121 Ferrara, Italy; erika.rimondi@unife.it (E.R.); elisabetta.melloni@unife.it (E.M.); paola.secchiero@unife.it (P.S.); giada.lodi@unife.it (G.L.)
[5] Department of Translational Medicine, University of Ferrara, 44121 Ferrara, Italy; marta.manfredini@unife.it (M.M.); annalisa.marcuzzi@unife.it (A.M.)
[6] Council for Agricultural Research and Economics (CREA), 00184 Rome, Italy
[7] Department of Environmental and Prevention Sciences, University of Ferrara, 44121 Ferrara, Italy
* Correspondence: crvsrg@unife.it; Tel.: +39-3333-989-690

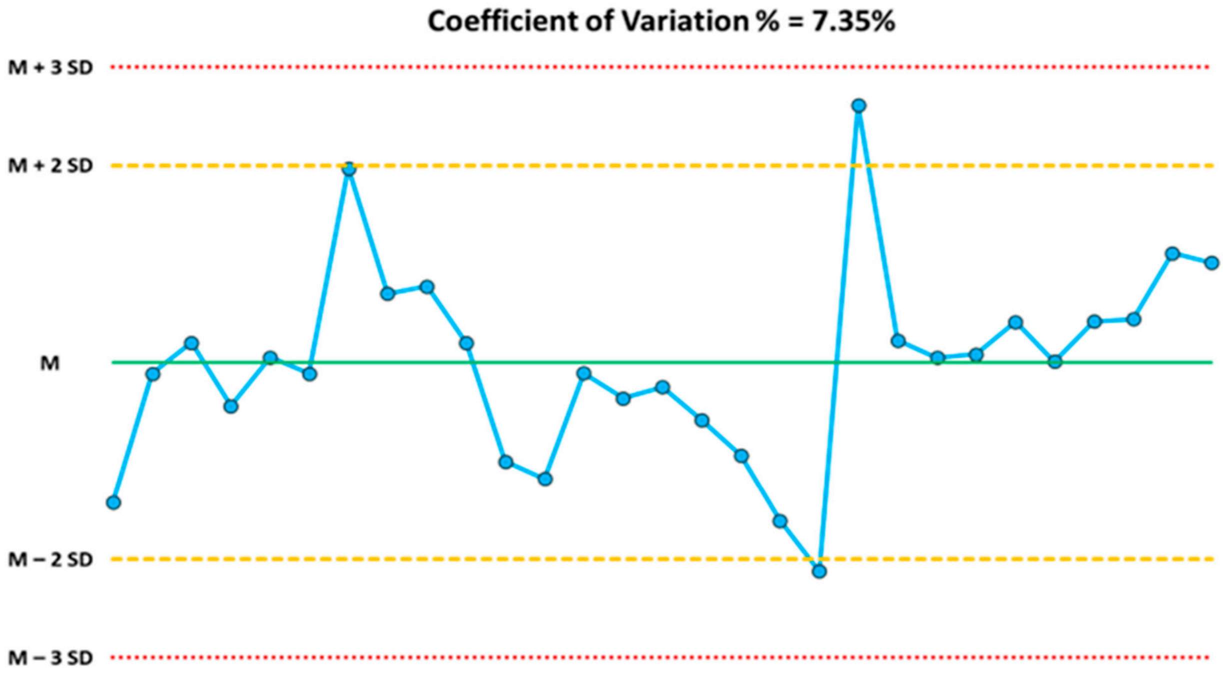

Figure S1. Control chart of the response of the internal standard (tridecane) along the analysis batch. The internal standard was preloaded on the fiber before sample extraction. An overall 7.35% coefficient of variation was obtained.

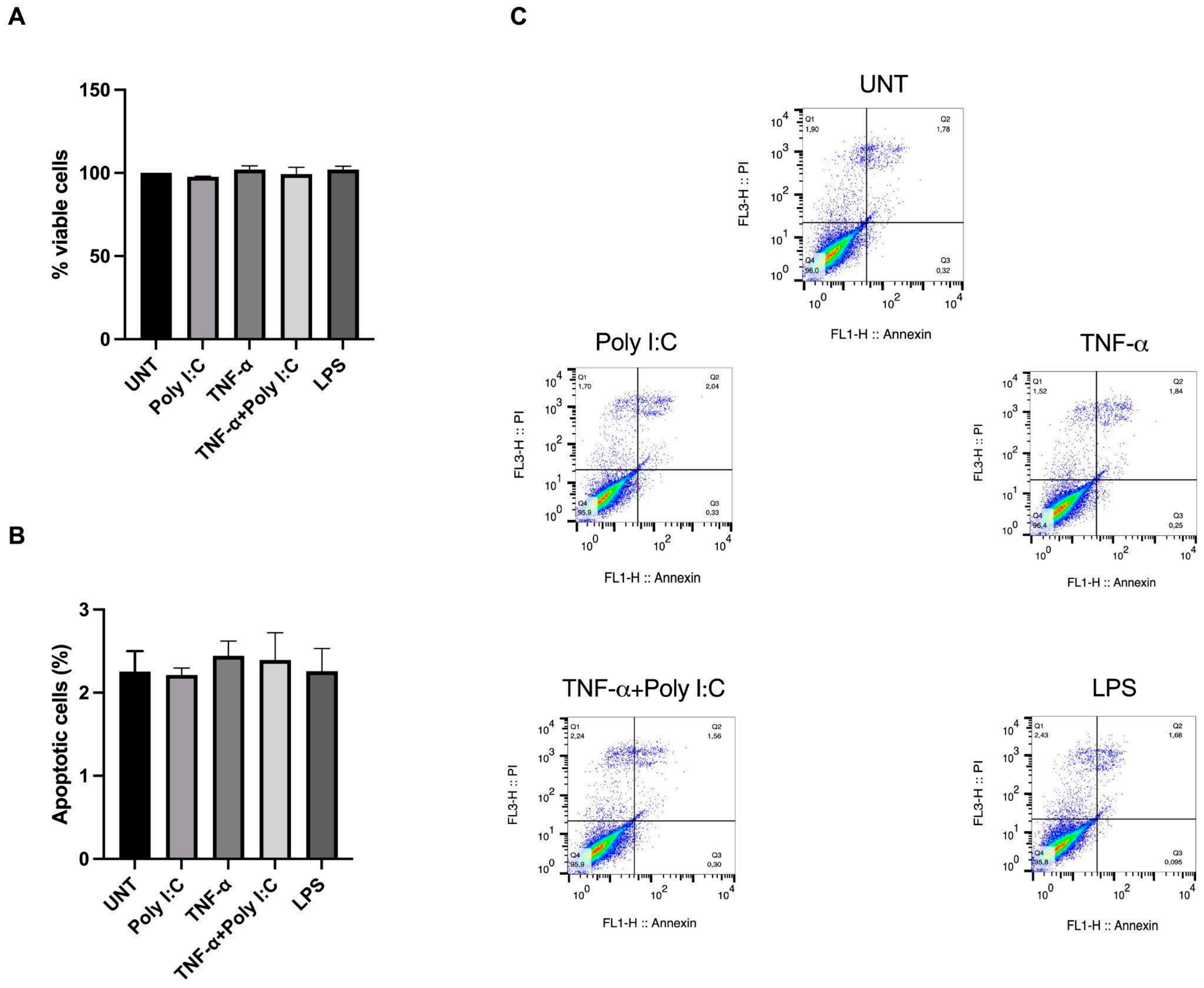

Figure S2. Effects of TNF-α, Poly I:C, and LPS treatments on cell viability and apoptosis in NHDF-c cells. Cells were treated with TNF-α, Poly I:C, TNF-α + Poly I:C, or LPS. Cell viability was assessed using the Trypan Blue exclusion (A) and the percentage of apoptotic cells (B) were assessed 6 h after treatment by flow cytometry. Representative flow cytometry dot plots are shown in panel (C). Results are presented as mean ± SEM from at least three independent experiments. Statistical analysis was performed using one-way ANOVA followed by Bonferroni's post hoc test. ***$p \leq 0.001$.

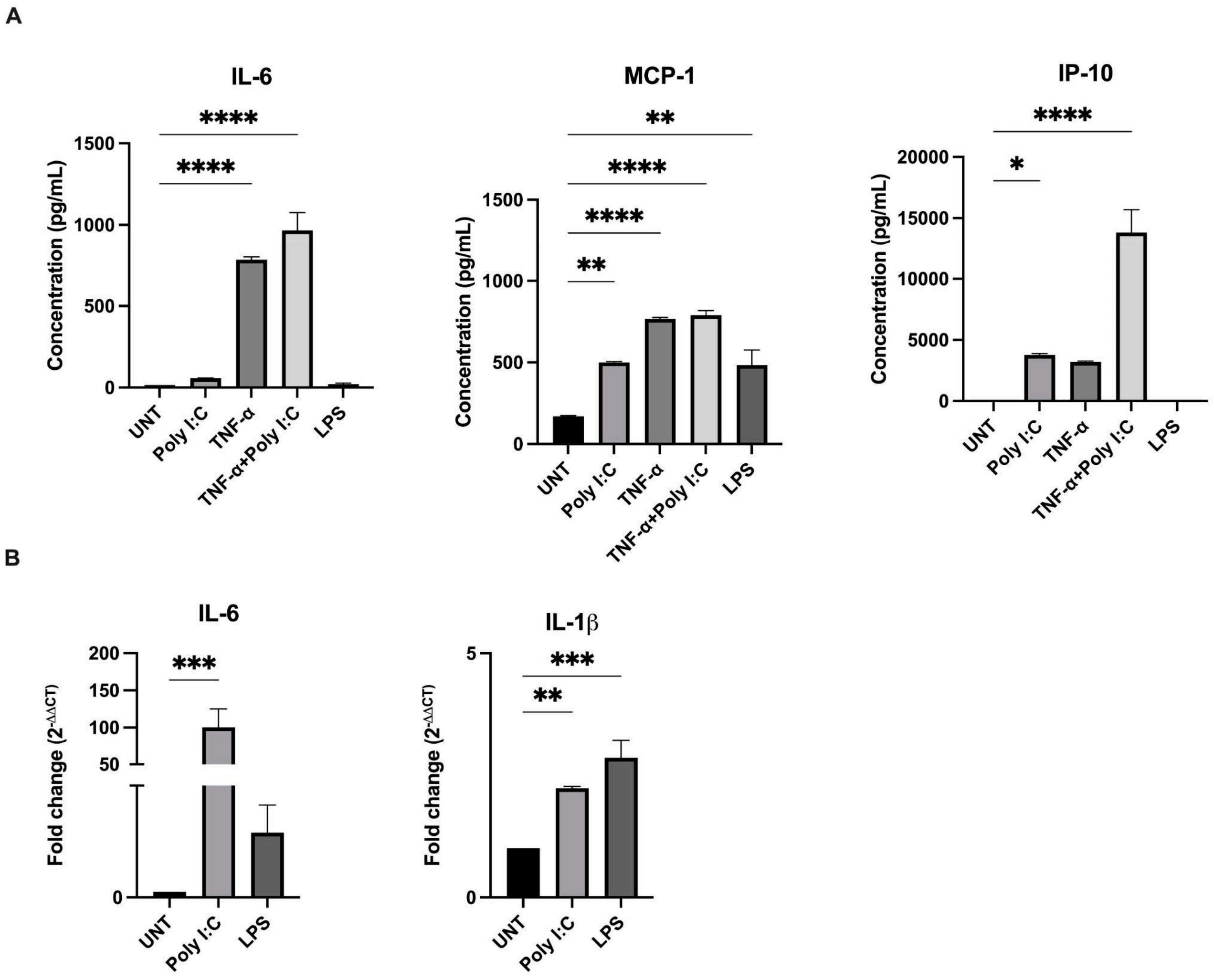

Figure S3. Effects of Poly I:C and LPS on cytokine production and gene expression in NHDF-c cells. NHDF-c cells were treated with TNF-α, Poly I:C, TNF-α + Poly I:C, or LPS, and inflammatory responses were evaluated. (A) Cytokine levels (IL-6, MCP-1, and IP-10) were measured in cell culture supernatants using Luminex multiplex analysis. (B) Relative gene expression levels of IL-6 and IL-1ß were determined by quantitative PCR in Poly I:C and LPS treatments. Data are presented as column bar graphs showing mean ± SEM from at least three independent experiments. Statistical analysis was performed using ordinary one-way ANOVA followed by Bonferroni's post hoc test. *$p < 0.05$, **$p < 0.01$, ***$p < 0.001$.

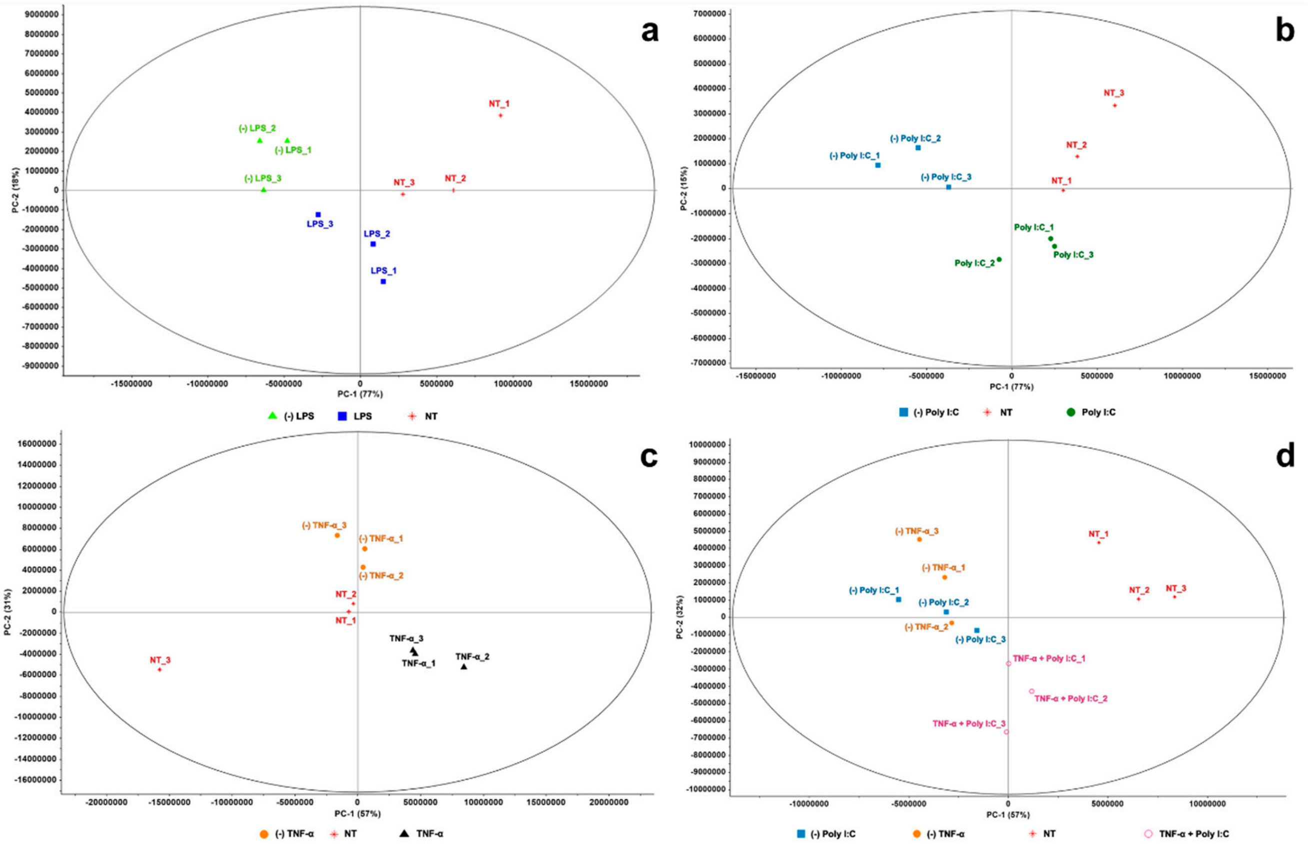

Figure S4a-d. PCA scores plot using selected significant features within each group after statistical filtering showing the distinct clustering of each treatment against the related controls. a) PCA performed on 49 fts for LPS, NT, (-) LPS; b) PCA performed on 23 fts for Poly I:C, NT, (-) Poly I:C; c) PCA performed on 55 fts for TNF-α, NT, (-) TNF-α; d) PCA performed on 55 fts for TNF-α + Poly I:C, NT, (-) Poly I:C, (-) TNF-α.

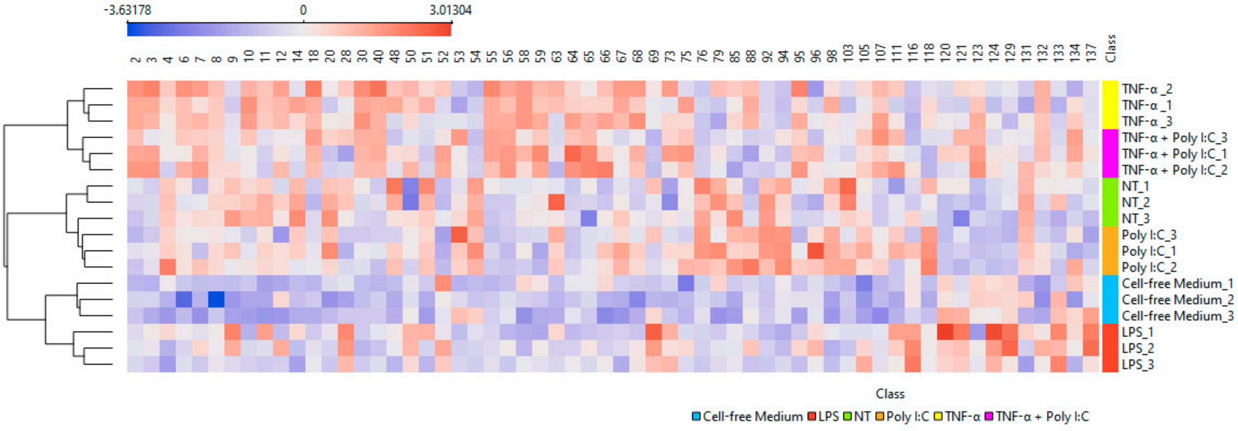

Figure S5. Heatmap of the selected feature with CV<30% within each group of treatment. Autoscaling procedures were applied to columns, while rows were clustered using Euclidean distance and average linkage. For compound ID, please refer to Table S1.

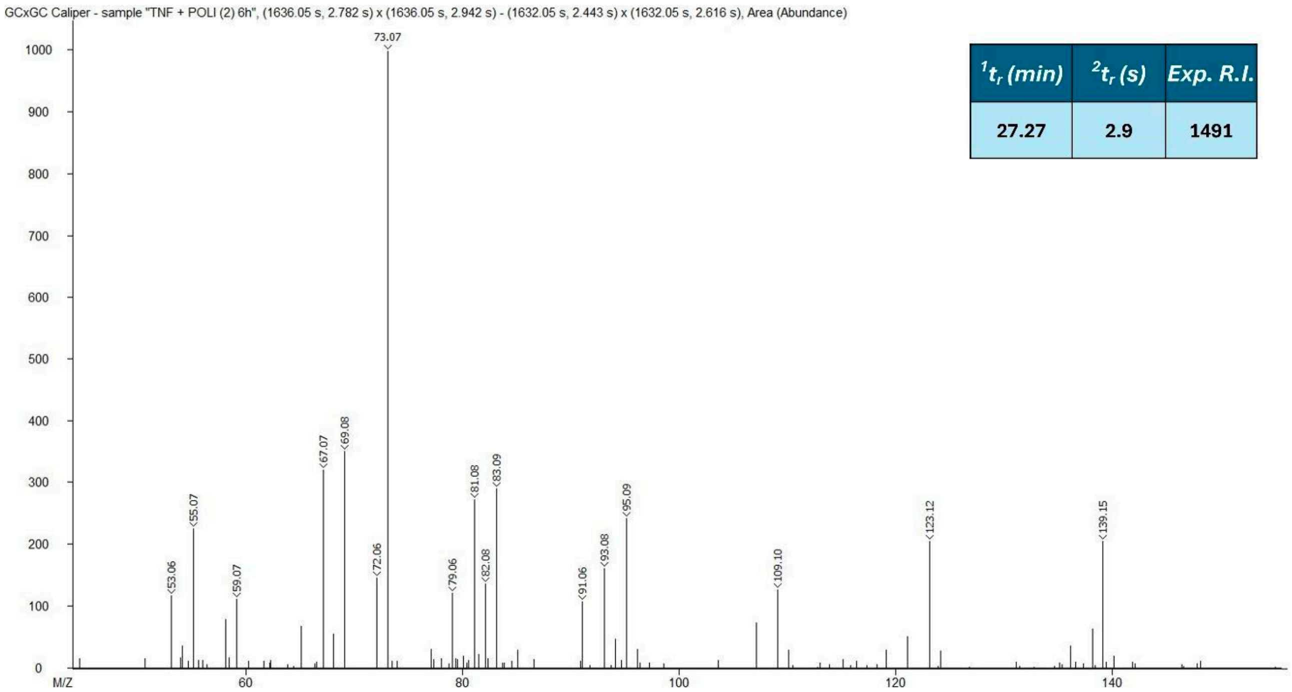

Figure S6. Representative GC×GC Caliper Mass Spectrum of the unknown volatile feature (Unknown 55), which was consistently altered across all inflammatory treatments, extracted from a TNF-*α* + Poly I:C-treated fibroblast sample (TNF-*α* + Poly I:C_2). Shown in the upper right corner is a table with its retention times in the first, $^{1}t_r$ *(min)*, and second, $^{2}t_r$ *(s)*, column and the calculated retention index (Exp. R.I.).

Table S1. List of 146 selected compounds with putative identification. $^{1}t_r$ (min): Retention time in the first dimension; $^{2}t_r$: retention time in the second dimension; Sim.: Spectral similarity; Quant. Ion: Quantification ion. Exp. R.I.: Experimental retention index values. Lib. R.I.: Library retention index values. Significance to stimulus controls using Mann–Whitney U test using the Wilcox-test function (refer to Figure 2 of the data analysis workflow). Compounds with equal putative library hits were grouped in the same chemical class and labelled as: "Alcohol", "Ether", "Hydrocarbon", and "Ester". NS: not significant

								Significance to stimulus controls (p-value)			
#	Compound name	$^{1}t_r$ (min)	$^{2}t_r$ (s)	Sim.	Quant. Ion	Exp. R.I.	Lib. R.I.	LPS	Poly I:C	TNF-α	TNF-α + Poly I:C
1	Unknown 1	3.33	1.71		60	758		NS	NS	0.02	0.04
2	Alcohol-1	3.33	1.72	862	69	758	765	NS	NS	0.02	NS
3	1-Pentanol	3.93	1.79	885	70	780	765	NS	NS	0.02	0.01
4	Heptane. 2.4-dimethyl-	5.07	1.48	856	85	821	821	NS	NS	0.02	0.02
5	Unknown 2	6.33	1.14		132	867		NS	NS	0.02	NS
6	Pent-3-enylamine	6.41	2.14	721	56	870		NS	NS	0.02	NS
7	1-Hexanol	6.40	2.15	860	84	870	868	NS	NS	NS	0.01
8	Unknown 3	6.47	2.2		56	872		0.02	NS	0.02	NS
9	Unknown 4	6.80	1.83		57	884		NS	NS	NS	0.01

10	2-Buten-1-ol. (Z)-	6.80	1.85	754	72	884		NS	NS	0.02	NS
11	1-Butanesulfonyl chloride	6.80	1.85	736	130	884		NS	0.02	NS	NS
12	Cyclohexanol	6.80	2.74	835	82	885	880	NS	NS	0.02	NS
13	Unknown 5	6.93	2.44		98	890		NS	0.02	NS	NS
14	Unknown 6	7.07	1.84		57	894		NS	0.02	NS	0.01
15	Ether-1	7.07	2.53	798	58	894		NS	NS	0.02	NS
16	Unknown 7	7.14	1.1		132	896		0.02	NS	NS	NS
17	Ester-1	8.73	2.5	902	55	945		0.02	NS	NS	NS
18	1-Heptanol	9.67	2.46	888	69	972	970	NS	0.02	0.02	0.01
19	Unknown 8	9.87	1.83		101	978		NS	0.02	0.02	NS
20	Unknown 9	9.87	1.83		45	978		NS	NS	NS	0.02
21	Unknown 10	9.95	2.47		43	980		NS	NS	NS	0.02
22	Propanoic acid. 2-hydroxy-. propyl ester	9.94	2.5	880	57	980		0.02	NS	NS	NS
23	Unknown 11	10.07	1.1		132	984		0.02	NS	NS	NS
24	4.5-Nonadiene	10.14	2.31	762	96	986		NS	NS	0.02	NS
25	Unknown 12	10.34	2.12		93	992		NS	NS	0.02	0.01
26	Diisobutyl cellosolve	10.40	2.56	867	58	994		NS	NS	0.02	NS
27	Unknown 13	10.42	2.68		105	994		NS	0.02	NS	NS
28	Unknown 14	10.60	1.2		73	999		0.02	NS	NS	NS
29	Decane. 2.6.7-trimethyl-	11.47	1.8	881	113	1024		0.02	NS	NS	NS
30	p-Cymene	11.48	2.74	818	119	1024	1025	NS	NS	0.02	0.01
31	1-Hexanol. 2-ethyl-	11.67	2.5	911	83	1030	1030	NS	NS	0.02	NS
32	Unknown 15	11.67	2.44		93	1030		NS	NS	0.02	NS
33	2-Propanone. 1-methoxy-	11.73	1.04	701	58	1031		NS	NS	NS	0.01
34	Unknown 16	11.73	2.6		154	1032		0.02	NS	NS	NS
35	Unknown 17	12.73	2.49		93	1059		NS	NS	NS	0.01
36	Unknown 18	12.80	2.26		56	1061		NS	NS	0.02	NS
37	Oxetane. 3.3-dimethyl-	13.16	2.67	747	56	1072		NS	0.02	0.02	0.01
38	Oxalic acid. isobutyl nonyl ester	13.53	2.13	826	85	1081		NS	0.02	NS	NS
39	1-Allyl-cyclopropanecarboxylic acid. 2.6-di-t-butyl-4-methyl-phenyl ester	13.93	0.2	725	109	1092		0.02	NS	NS	NS
40	Linalool	14.23	2.56	735	93	1100	1099	NS	NS	0.02	0.02
41	1-Hexanol. 3-methyl-	14.29	2.46	763	55	1102		NS	NS	0.02	NS
42	Unknown 19	14.26	2.52		69	1102		NS	NS	NS	0.04
43	Unknown 20	14.25	2.55		93	1102		NS	NS	0.02	NS
44	7-Oxabicyclo[4.1.0]heptane. 3-oxiranyl-	14.40	2.66	818	124	1106		NS	0.02	NS	0.02
45	Cyclobutene. 2-propenylidene-	14.77	0.21	860	91	1114		NS	0.02	NS	0.01
46	Benzene. (propoxymethyl)-	14.77	0.22	849	91	1114		NS	NS	NS	0.01
47	Unknown 21	14.87	1.0		132	1118		0.02	NS	NS	NS
48	Unknown 22	14.97	1.7		126	1120		0.02	NS	NS	NS
49	Unknown 23	15.21	2.6		57	1128		0.02	NS	NS	NS
50	6-Methyl-1-octanol	15.81	2.6	811	97	1145	1144	0.02	NS	NS	NS
51	Unknown 24	16.13	1.57		73	1154		NS	NS	NS	0.01
52	Unknown 25	16.40	1.5		297	1161		0.02	NS	NS	NS
53	Menthol	16.80	2.92	890	138	1173	1175	NS	0.02	NS	NS
54	1-Octene. 3.7-dimethyl-	16.87	2.6	811	70	1175		0.02	NS	NS	NS
55	Terpinen-4-ol	17.00	3.12	754	111	1179	1177	NS	NS	0.02	0.01

56	Unknown 26	17.00	3.14		119	1179		NS	NS	NS	0.01
57	Unknown 27	17.43	3.31		93	1192		NS	NS	NS	0.04
58	α-Terpineol	17.47	3.31	789	136	1192	1189	NS	NS	0.02	NS
59	Unknown 28	17.47	3.32		79	1192		NS	NS	0.02	0.01
60	Ethanone. 1-cyclopropyl-	17.94	3.85	831	69	1206		NS	NS	NS	0.02
61	Unknown 29	18.16	3.12		81	1211		NS	0.02	NS	NS
62	Acetic acid. [(1.1-dimethylethyl)thio]-	18.14	3.2	819	57	1211		0.02	NS	NS	NS
63	Unknown 30	18.27	1.59		73	1214		NS	NS	NS	0.01
64	Unknown 31	18.27	3.13		69	1215		NS	NS	0.02	0.01
65	Unknown 32	18.27	3.13		115	1215		NS	NS	NS	0.01
66	Unknown 33	18.33	0.36		102	1216		NS	NS	0.02	NS
67	Unknown 34	18.33	0.36		135	1216		NS	NS	0.02	NS
68	Unknown 35	18.33	0.36		132	1216		NS	NS	0.02	0.04
69	Unknown 36	18.47	2.5		326	1221		0.02	NS	NS	NS
70	Unknown 37	19.08	2.04		269	1238		NS	NS	0.02	NS
71	Unknown 38	19.14	2.7		125	1240		0.02	NS	NS	NS
72	Cyclopentanemethanol	19.17	2.3	742	68	1242		NS	0.02	NS	NS
73	Hydrocarbon-1	19.36	1.8	801	71	1246		0.02	NS	NS	NS
74	(3.3-Dimethyloxiranyl)methanol	19.33	2.88	763	59	1246		NS	NS	0.02	NS
75	Unknown 39	19.73	3.11		93	1258		NS	NS	NS	0.01
76	Unknown 40	19.93	1.6		73	1263		0.02	NS	0.02	0.04
77	Ester-2	20.14	2.87	939	59	1269		NS	NS	NS	0.02
78	2-Propenoic acid. ethenyl ester	20.20	1.7	902	55	1271		0.02	NS	NS	NS
79	Heptadecane. 2.6-dimethyl-	20.34	1.8	875	71	1275		NS	NS	NS	0.01
80	Unknown 41	20.47	0.08		66	1278		NS	0.02	NS	NS
81	1-Octanol. 2-butyl-	20.60	1.77	863	113	1282	1277	NS	NS	0.02	0.02
82	Hydroxymethyl 2-hydroxy-2-methylpropionate	20.67	0.31	939	59	1284		NS	NS	NS	0.01
83	Unknown 42	20.65	2.63		57	1285		NS	NS	0.02	NS
84	Unknown 43	20.74	0.5		131	1286		0.02	NS	NS	NS
85	Butyl 2-ethylhexanoate	21.00	2.5	866	145	1294		0.02	NS	NS	NS
86	Unknown 44	22.20	1.0		132	1330		0.02	NS	NS	NS
87	Unknown 45	22.80	2.01		70	1349		NS	NS	0.02	NS
88	Di-tert-Butyl ether	23.00	2.68	741	115	1355		NS	NS	0.02	NS
89	Unknown 46	23.13	1.0		132	1359		0.02	NS	0.02	NS
90	p-Isopropylphenetole	23.33	3.6	728	149	1366		0.02	NS	NS	NS
91	Unknown 47	23.42	2.72		57	1368		NS	NS	0.02	NS
92	Unknown 48	23.87	3.65		56	1382		NS	NS	0.02	NS
93	1.2-Ethanediol. monobenzoate	24.06	3.54	857	123	1388		NS	NS	0.02	NS
94	Ether-2	24.20	1.71	761	73	1392		NS	0.02	NS	NS
95	Phenol. 4-(1.1-dimethylpropyl)-	24.40	3.75	858	135	1399	1400	NS	NS	0.02	0.01
96	Dodecanal	24.80	2.75	908	82	1411	1409	NS	0.02	NS	NS
97	Unknown 49	25.02	3.5		154	1418		0.02	NS	NS	0.01
98	Unknown 50	25.27	1.48		73	1425		NS	0.02	NS	NS
99	Unknown 51	25.94	2.62		131	1448		NS	NS	NS	0.04
100	Unknown 52	26.20	1.5		73	1453		NS	NS	0.02	NS
101	Oxalic acid. isohexyl neopentyl ester	26.22	1.95	722	71	1456		NS	NS	0.02	NS

102	1.4-Hexadiene. 3.3.5-trimethyl-	26.20	2.88	709	109	1456		NS	NS	0.02	0.01
103	Unknown 53	26.26	1.5		73	1458		NS	NS	0.02	0.01
104	Sulfurous acid. 2-ethylhexyl hexyl ester	26.47	1.92	785	85	1464		0.02	NS	NS	0.01
105	2.5-Cyclohexadiene-1.4-dione. 2.6-bis(1.1-dimethylethyl)-	26.60	3.18	874	220	1469	1472	NS	0.02	NS	NS
106	Unknown 54	26.93	3.83		57	1481		NS	NS	NS	0.01
107	Unknown 55	27.27	2.9		73	1491		0.02	0.02	0.02	0.01
108	1H-Indene. 1-chloro-2.3-dihydro-	27.54	0.24	830	116	1498		NS	NS	0.02	0.01
109	Unknown 56	27.54	1.5		73	1499		0.02	NS	NS	NS
110	Cyclopropane. 1-butyl-2-pentyl-. trans-	27.60	2.0	767	69	1501		0.02	NS	NS	NS
111	2.4-Di-tert-butylphenol	28.00	3.23	905	191	1516	1514	0.02	NS	NS	0.01
112	Unknown 57	28.00	3.2		159	1516		0.02	NS	NS	NS
113	Unknown 58	28.07	1.95		71	1517		NS	NS	NS	0.01
114	1.3-Dioxolane-2-methanol	28.62	1.64	847	73	1535		NS	NS	0.02	NS
115	Unknown 59	28.64	3.11		135	1538		NS	NS	NS	0.04
116	Undecane. 4.7-dimethyl-	28.86	1.9	824	85	1544		0.02	NS	NS	NS
117	Octane. 5-ethyl-2-methyl-	29.14	2.0	707	85	1554		0.02	NS	NS	NS
118	Unknown 60	29.34	1.67		73	1560		NS	0.02	NS	NS
119	1.2.4-Triazolo[4.3-b]pyridazine	29.80	3.6	703	120	1577		0.02	NS	NS	NS
120	Unknown 61	30.14	2.1		70	1588		0.02	NS	NS	NS
121	Cetene	30.34	2.1	784	97	1595	1592	0.02	NS	NS	NS
122	Unknown 62	30.42	1.5		147	1597		0.02	NS	NS	NS
123	Unknown 63	30.47	0.81		149	1598		NS	NS	NS	0.01
124	Unknown 64	30.54	2.0		83	1601		0.02	NS	NS	NS
125	Unknown 65	31.47	0.9		132	1635		0.02	NS	NS	NS
126	Unknown 66	31.54	2.01		71	1638		NS	NS	NS	0.04
127	Decane. 6-ethyl-2-methyl-	31.81	2.0	751	71	1647		0.02	NS	NS	NS
128	1.3-Dioxolane. 2-hexyl-	32.00	1.57	761	73	1654		NS	0.02	NS	NS
129	3-Ethoxyacrylonitrile	32.07	2.3	844	68	1657		0.02	NS	NS	NS
130	Ester-3	32.33	2.0	708	71	1666		0.02	NS	NS	NS
131	n-Hexyl salicylate	32.67	3.54	792	120	1679	1682	NS	0.02	NS	0.01
132	2H-Pyran. 2-(bromomethyl)tetrahydro-	32.80	0.13	778	85	1682		0.02	NS	0.02	0.01
133	2.4.6.8-Tetramethyl-1-undecene	33.13	2.2	766	97	1695		0.02	NS	NS	NS
134	Phenol. 2-(phenylmethoxy)-	34.18	2.65	769	91	1736		NS	NS	NS	0.04
135	Sulfurous acid. isobutyl pentyl ester	34.50	2.02	718	71	1746		NS	NS	NS	0.04
136	Unknown 67	34.85	1.97		57	1761		NS	NS	0.02	NS
137	Glycine. N-(3-methyl-1-oxo-2-butenyl)-. methyl ester	34.87	2.4	734	83	1761		0.02	NS	NS	NS
138	2-Amino-4-(1-ethylpropyl)-4H-benzo[h]chromene-3-carbonitrile	35.34	1.46	710	73	1778		NS	NS	0.02	NS
139	Unknown 68	35.47	2.46		57	1784		NS	NS	NS	0.02
140	1H-1.2.4-Triazole-3-carboxaldehyde. 5-methyl-	35.47	2.93	755	83	1784		NS	0.02	NS	NS
141	Ester-4	36.20	1.97	703	71	1812		NS	NS	NS	0.01
142	Acetic acid. (dodecahydro-7-hydroxy-1.4b.8.8-	36.27	2.56	715	71	1815		NS	NS	0.02	NS

	tetramethyl-10-oxo-2(1H)-phenanthrenylidene)-.2-(dimethylamino)ethyl ester									
143	Unknown 69	37.07	2.1		57	1847	0.02	NS	NS	NS
144	Unknown 70	37.40	1.5		147	1860	0.02	NS	NS	NS
145	Z-2-Dodecenol	37.87	2.92	730	71	1879	NS	NS	0.02	NS
146	Unknown 71	38.14	1.45		151	1889	NS	NS	0.02	NS

Table S2. Log2FC of the selected feature for LPS treatment compared to non-treated cell controls (NT) and LPS control stimulus solution ((-)LPS).

#	Compound name	Log2FC LPS against NT	Log2FC LPS against (-)LPS
8	Unknown 3	-0.17	0.47
16	Unknown 7	1.77	1.23
17	Ester-1	0.99	0.48
22	Propanoic acid. 2-hydroxy-. propyl ester	1.13	0.59
23	Unknown 11	1.32	1.79
28	Unknown 14	0.23	0.52
29	Decane. 2.6.7-trimethyl-	0.40	0.88
34	Unknown 16	-0.53	0.90
39	1-Allyl-cyclopropanecarboxylic acid. 2.6-di-t-butyl-4-methyl-phenyl ester	-1.71	12.82
47	Unknown 21	2.05	2.30
48	Unknown 22	-0.21	0.26
49	Unknown 23	1.73	1.22
50	(S)-(+)-6-Methyl-1-octanol	0.75	0.46
52	Unknown 25	0.78	0.71
54	1-Octene. 3.7-dimethyl-	-0.89	0.66
62	Acetic acid. [(1.1-dimethylethyl)thio]-	2.73	1.58
69	Unknown 36	0.58	1.17
71	Unknown 38	5.03	1.75
73	Hydrocarbon-1	1.04	0.86
76	Unknown 40	-1.38	0.51
78	2-Propenoic acid. ethenyl ester	3.23	1.95
84	Unknown 43	1.54	1.03
85	Butyl 2-ethylhexanoate	-0.14	0.04
86	Unknown 44	1.08	1.53
89	Unknown 46	0.57	2.03
90	p-Isopropylphenetole	2.15	1.74
97	Unknown 49	1.02	0.47
104	Sulfurous acid. 2-ethylhexyl hexyl ester	0.54	0.81
107	Unknown 55	0.33	1.79
109	Unknown 56	1.66	1.48
110	Cyclopropane. 1-butyl-2-pentyl-. trans-	0.91	0.56
111	2.4-Di-tert-butylphenol	0.24	0.24
112	Unknown 57	0.55	1.61
116	Undecane. 4.7-dimethyl-	0.68	0.70

117	Octane. 5-ethyl-2-methyl-	1.71	2.00
119	1.2.4-Triazolo[4.3-b]pyridazine	0.73	1.10
120	Unknown 61	0.73	0.61
121	Cetene	1.11	0.83
122	Unknown 62	1.62	0.44
124	Unknown 64	1.02	0.87
125	Unknown 65	1.44	2.11
127	Decane. 6-ethyl-2-methyl-	1.91	1.46
129	3-Ethoxyacrylonitrile	0.98	0.70
130	Ester-3	0.92	1.19
132	2H-Pyran. 2-(bromomethyl)tetrahydro-	0.37	1.01
133	2.4.6.8-Tetramethyl-1-undecene	0.73	1.92
137	Glycine. N-(3-methyl-1-oxo-2-butenyl)-. methyl ester	0.97	0.82
143	Unknown 69	2.03	2.33
144	Unknown 70	0.60	0.53

Table S3. Log2FC of the selected feature for Poly I:C treatment compared to non-treated cell controls (NT) and Poly I:C control stimulus solution ((-) Poly I:C).

#	Compound name	Log2FC Poly I:C against NT	Log2FC Poly I:C against (-)Poly I:C
11	1-Butanesulfonyl chloride	-0.07	0.14
13	Unknown 5	1.08	1.12
14	Unknown 6	-0.16	0.26
18	1-Heptanol	0.36	1.33
19	Unknown 8	0.90	0.96
27	Unknown 13	0.69	0.18
37	Oxetane. 3.3-dimethyl-	0.22	0.92
38	Oxalic acid. isobutyl nonyl ester	0.47	1.07
44	7-Oxabicyclo[4.1.0]heptane. 3-oxiranyl-	0.53	0.57
45	Cyclobutene. 2-propenylidene-	0.95	1.27
53	Levomenthol	0.32	0.58
61	Unknown 29	1.62	5.76
72	Cyclopentanemethanol	0.71	0.87
80	Unknown 41	-0.34	4.14
94	Ether-2	0.20	0.47
96	Dodecanal	0.35	0.37
98	Unknown 50	0.13	0.48
105	2.5-Cyclohexadiene-1.4-dione. 2.6-bis(1.1-dimethylethyl)-	0.24	0.88
107	Unknown 55	0.69	2.66
118	Unknown 60	0.35	0.65
128	Cyclic acetal-2	0.91	0.88
131	n-Hexyl salicylate	-0.08	0.57
140	1H-1.2.4-Triazole-3-carboxaldehyde. 5-methyl-	0.17	1.27

Table S4. Log2FC of the selected feature for TNF treatment compared to non-treated cell controls (NT) and NTF control stimulus solution ((-) TNF-*α*).

#	Compound name	Log2FC TNF-α against NT	Log2FC TNF-α against (-)TNF-α
1	Unknown 1	0.53	1.45
2	Alcohol-1	0.62	0.14
3	1-Pentanol	0.50	0.75
4	Heptane. 2.4-dimethyl-	-0.06	0.34
5	Unknown 2	1.96	0.94
6	Pent-3-enylamine	0.46	1.25
8	Unknown 3	0.11	0.69
10	2-Buten-1-ol. (Z)-	0.06	0.25
12	Cyclohexanol	0.13	0.16
15	Ether-1	1.01	2.04
18	1-Heptanol	1.05	1.92
19	Unknown 8	0.92	1.41
24	4.5-Nonadiene	0.86	1.53
25	Unknown 12	1.12	0.37
26	Diisobutyl cellosolve	0.82	1.63
30	p-Cymene	0.52	0.30
31	1-Hexanol. 2-ethyl-	0.36	0.10
32	Unknown 15	3.96	1.05
36	Unknown 18	0.22	0.19
37	Oxetane. 3.3-dimethyl-	0.56	0.70
40	Linalool	0.74	0.34
41	1-Hexanol. 3-methyl-	0.78	3.48
43	Unknown 20	0.74	0.33
55	Terpinen-4-ol	0.71	0.25
58	α-Terpineol	0.39	0.49
59	Unknown 28	0.33	0.23
64	Unknown 31	0.57	0.45
66	Unknown 33	0.29	0.54
67	Unknown 34	0.30	0.74
68	Unknown 35	0.45	0.57
70	Unknown 37	2.09	0.48
74	(3.3-Dimethyloxiranyl)methanol	0.35	0.52
76	Unknown 40	-1.47	0.48
81	1-Octanol. 2-butyl-	-0.30	0.45
83	Unknown 42	1.10	5.73
87	Unknown 45	1.17	0.80
88	Di-tert-Butyl ether	0.47	0.55
89	Unknown 46	1.01	1.29
91	Unknown 47	0.18	0.11
92	Unknown 48	-1.31	0.25
93	1.2-Ethanediol. monobenzoate	-0.55	0.78
95	Phenol. 4-(1.1-dimethylpropyl)-	0.37	0.48
100	Unknown 52	-1.66	1.48
101	Oxalic acid. isohexyl neopentyl ester	0.85	0.95

102	1.4-Hexadiene. 3.3.5-trimethyl-	0.70	7.51
103	Unknown 53	-0.67	0.28
107	Unknown 55	1.06	2.55
108	1H-Indene. 1-chloro-2.3-dihydro-	0.32	0.39
114	1.3-Dioxolane-2-methanol	0.90	0.54
132	2H-Pyran. 2-(bromomethyl)tetrahydro-	0.56	1.62
136	Unknown 67	1.36	0.64
138	2-Amino-4-(1-ethylpropyl)-4H-benzo[h]chromene-3-carbonitrile	0.52	1.06
142	Acetic acid. (dodecahydro-7-hydroxy-1.4b.8.8-tetramethyl-10-oxo-2(1H)-phenanthrenylidene)-.2-(dimethylamino)ethyl ester	0.86	0.98
145	Z-2-Dodecenol	0.62	7.09
146	Unknown 71	1.30	5.91

Table S5. Log2FC of the selected feature for TNF-*α* + Poly I:C treatment compared to non-treated cell controls (NT) and TNF-*α* + Poly I:C control stimulus solution ((-) TNF-*α* + Poly I:C).

#	Compound name	Log2FC TNF-α + Poly I:C vs NT	Log2FC TNF-α + Poly I:C vs (-)TNF-α + Poly I:C
1	Unknown 1	0.42	0.66
3	1-Pentanol	0.40	0.62
4	Heptane. 2.4-dimethyl-	-0.19	0.17
7	1-Hexanol	0.54	1.91
9	Unknown 4	-0.15	0.18
14	Unknown 6	-0.27	0.14
18	1-Heptanol	0.97	1.88
20	Unknown 9	-0.37	0.45
21	Unknown 10	0.92	1.14
25	Unknown 12	1.35	0.78
30	p-Cymene	0.40	0.25
33	2-Propanone. 1-methoxy-	0.79	1.76
35	Unknown 17	0.92	0.61
37	Oxetane. 3.3-dimethyl-	0.52	0.91
40	Linalool	0.63	0.17
42	Unknown 19	0.92	0.24
44	7-Oxabicyclo[4.1.0]heptane. 3-oxiranyl-	0.29	0.33
45	Cyclobutene. 2-propenylidene-	1.57	1.76
46	Benzene. (propoxymethyl)-	0.88	1.78
51	Unknown 24	-0.32	0.26
55	Terpinen-4-ol	0.64	0.21
56	Unknown 26	0.83	0.21
57	Unknown 27	6.02	0.31
59	Unknown 28	0.41	0.56
60	Ethanone. 1-cyclopropyl-	2.07	1.70
63	Unknown 30	-0.20	0.17
64	Unknown 31	0.81	0.54
65	Unknown 32	1.17	0.78

68	Unknown 35	0.30	0.27
75	Unknown 39	0.50	2.22
76	Unknown 40	-1.43	0.37
77	Ester- 2	1.06	0.53
79	Heptadecane. 2.6-dimethyl-	-0.25	0.37
81	1-Octanol. 2-butyl-	-0.46	0.37
82	Hydroxymethyl Hydroxylated ester-2	1.68	3.01
95	Phenol. 4-(1.1-dimethylpropyl)-	0.34	0.43
97	Unknown 49	1.43	0.55
99	Unknown 51	0.89	1.68
102	1.4-Hexadiene. 3.3.5-trimethyl-	0.63	7.55
103	Unknown 53	-0.55	0.38
104	Sulfurous acid. 2-ethylhexyl hexyl ester	0.27	0.75
106	Unknown 54	-0.55	3.11
107	Unknown 55	1.13	2.84
108	1H-Indene. 1-chloro-2.3-dihydro-	0.42	0.84
111	2.4-Di-tert-butylphenol	0.26	0.31
113	Unknown 58	0.74	0.92
115	Unknown 59	-1.51	0.79
123	Unknown 63	0.69	0.27
126	Unknown 66	0.71	0.54
131	n-Hexyl salicylate	-0.22	0.50
132	2H-Pyran. 2-(bromomethyl)tetrahydro-	0.47	1.69
134	Phenol. 2-(phenylmethoxy)-	0.45	0.26
135	Sulfurous acid. isobutyl pentyl ester	0.99	1.01
139	Unknown 68	3.00	1.27
141	Ester-2	0.41	0.46
